# Gravity and Known Size Calibrate Visual Information to Time Parabolic Trajectories

**DOI:** 10.3389/fnhum.2021.642025

**Published:** 2021-08-23

**Authors:** Borja Aguado, Joan López-Moliner

**Affiliations:** Vision and Control of Action (VISCA) Group, Department of Cognition, Development and Psychology of Education, Institut de Neurociències, Universitat de Barcelona, Barcelona, Spain

**Keywords:** 3D perception, calibration, internal models, optic flow, prior knowledge, TTC

## Abstract

Catching a ball in a parabolic flight is a complex task in which the time and area of interception are strongly coupled, making interception possible for a short period. Although this makes the estimation of time-to-contact (TTC) from visual information in parabolic trajectories very useful, previous attempts to explain our precision in interceptive tasks circumvent the need to estimate TTC to guide our action. Obtaining TTC from optical variables alone in parabolic trajectories would imply very complex transformations from 2D retinal images to a 3D layout. We propose based on previous work and show by using simulations that exploiting prior distributions of gravity and known physical size makes these transformations much simpler, enabling predictive capacities from minimal early visual information. Optical information is inherently ambiguous, and therefore, it is necessary to explain how these prior distributions generate predictions. Here is where the role of prior information comes into play: it could help to interpret and calibrate visual information to yield meaningful predictions of the remaining TTC. The objective of this work is: (1) to describe the primary sources of information available to the observer in parabolic trajectories; (2) unveil how prior information can be used to disambiguate the sources of visual information within a Bayesian encoding-decoding framework; (3) show that such predictions might be robust against complex dynamic environments; and (4) indicate future lines of research to scrutinize the role of prior knowledge calibrating visual information and prediction for action control.

## Introduction

Intercepting a ball in a parabolic trajectory before reaching ground level is a fundamental task in different sports: batting a baseball, hitting a high lob in tennis, or heading a football. In those situations, the time at which the interception is possible is very tight, yet our performance is astonishing. Time-to-contact (from now on TTC), that is, the time until an object reaches a location of interest, can provide very useful information that would help anticipate motor programs to solve those tasks.

In principle, to intercept a target, it would be enough to estimate its position and predict its future position based on speed estimates. Solutions based on this idea have been put forward for 2D motion (Kwon et al., 2015; Aguilar-Lleyda et al., 2018) but the generalization to 3D parabolic trajectories faces complex problems deeply rooted in the inverse-projection problem of Perception. The inverse problem of Perception refers to the ambiguous mapping between distal stimuli and final percept (Pizlo, 2001; Kersten et al., 2004). Unlike previous attempts where TTC is obtained from optical variables, in this article, we propose that some constants in the environment like gravity and size are considered and ease the otherwise complex transformation of optical variables to a 3D world to obtain relevant variables like TTC. The stance taken in this work will assume that we make implicit inferences (Von Helmholtz, 1867) about the present and future states of the world to act. However, the nature of the information guiding the control of action is an ongoing source of debate within the study of Perception.

## Two Theories for Interceptive Control

### Information-Based Control

**Figure 1:**
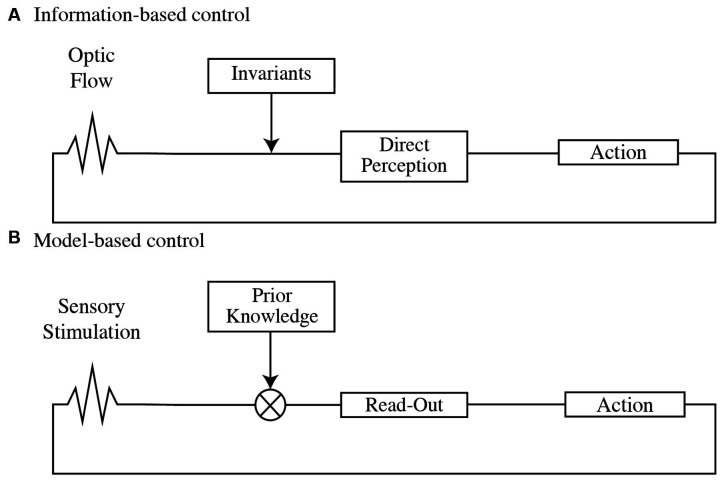
**(A)** Optic flow conforms to invariants that specify properties of the environment (direct perception), indicating the adequacy and availability of action within the task. **(B)** Sensory stimulation is combined with prior information to infer current or future states in the environment (read-out), providing the grounds to plan and adapt action.

The information-based control perspective, rooted in the Ecological or Gibsonian framework of Psychology (Gibson, 1966, 1979; Figure 1A), assumes that perceptual information is governed by certain physical regularities (Turvey et al., 1981) that can be captured and exploited to control our action. Under ecologically valid conditions (i.e., full-cue conditions), our perceptual system would be attuned to perceptual invariants directly specifying the characteristics of an event without the need to perform internal computations according to which humans act (Gibson, 1979). Thus, the information to solve a given task is directly specified in the optic flow (direct perception) explaining why only identifying task-relevant visual variables will determine an actor’s successful action from a perceptual perspective.

Under this framework, mainstream interpretations argue that the role of the observer is to actively seek out invariants within certain task-relevant pieces of optic information and unfold a coupled action based on instantaneous information. Following this line, detecting and maintaining invariant stimulation requires reducing the difference or the error with an “ideal value.” Because of that, these strategies are also called error-nulling strategies (Fajen, 2005b). Based on this idea, different control laws have been proposed for visually guided actions such as intercepting a moving object (Warren et al., 2001; Wilkie and Wann, 2003; Bruggeman et al., 2007; Zhao et al., 2019), braking (Lee, 1976) or catching a ball on the fly (Chapman, 1968; Michaels and Oudejans, 1992; McLeod and Dienes, 1993; McBeath et al., 1995).

Among the previous examples, catching a ball in a parabolic flight is a paradigmatic interception case including locomotion and manual interceptive phases. The study of the underlying mechanisms regarding the locomotive phase is usually referred to as the outfielder problem (Todd, 1981). The outfielder problem studies a case of interception in which baseball players known as outfielders must move to catch a high-flying ball in a parabolic trajectory before it hits the ground (Chapman, 1968; Todd, 1981; Michaels and Oudejans, 1992).

**Figure 2:**
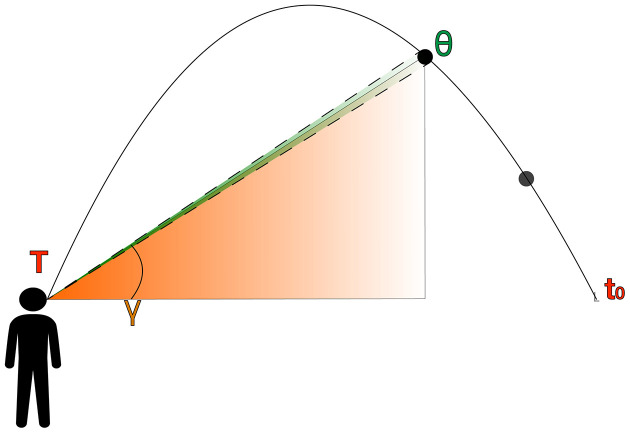
Lateral view of a parabolic trajectory depicting the primary primitive monocular cues, that is, retinal size (green projection; θ) and elevation angle (orange projection; γ).

The first catching error-nulling strategy put forward to explain action control within the outfielder problem was Chapman’s strategy (Chapman, 1968). S. Chapman noticed that when a ball follows a parabolic trajectory on a collision course with the observer, the elevation angle (γ; see Figure 2), that is, the vertical angle between the ball’s position and an observer’s eye level, increases during the whole trajectory. Therefore, all an observer would need to do to navigate towards the interception location is keeping the elevation angle increasing during the whole trajectory at a constant rate. However, others suggested it should increase at a decreasing rate (McLeod and Dienes, 1993, 1996).

Later, Michaels and Oudejans (1992) described Chapman’s strategy in terms of the projected image in the vertical plane at launch distance. If the ball is in a collision course with the viewer, the vertical projection of the ball increases linearly through the trajectory. In any other case, the image of the ball would displace non-linearly, that is, accelerated. Therefore, to catch a ball in a parabolic trajectory, one needs to actively maintain the acceleration of the projected vertical position of the ball at zero. Motivated by that, this strategy was named Optic Acceleration Cancellation (from now on, OAC) in McBeath et al. (1995).

Although the error-nulling strategies emerged within the ecological framework, they conflict with a key concept at the core of the Ecological theory, the theory of affordances (Gibson, 1979). The affordance-based theory emphasizes the idea that observers are tuned to the availability of an action given a sensory array. This tuning would be a by-product of a gauging process that maps optic into movement information and even into optic correlates in object size units (Peper et al., 1994; Jacobs and Michaels, 2006). Hence, if a fielder is correctly calibrated, acting to keep certain variables of interest into a “safe region” would ensure interception. This notion provides the grounds for an affordance-based control strategy theory (Fajen, 2007).

Reformulating the OAC under the scope of the affordance-based control, either canceling out vertical acceleration or running at maximum speed without being able to cancel out vertical acceleration, would be required to perceive catchability. However, a series of studies (Postma et al., 2017, 2018) found that actual catchers did not need to cancel out acceleration nor run at their maximum speed to judge catchability. These results cast doubts on the informational nature of a catchability affordance.

Despite a lesser dependence on immediate visual information, an affordance-based control strategy is still dependent on instantaneous visual information. In this respect, simulations of the locomotion based on error-nulling strategies were irreconcilable with actual catches when accounting for human neuromotor acceleration and sensorimotor integration delays (McLeod and Dienes, 1996; Kistemaker et al., 2006, 2009). Consequently, a minimal prediction seems necessary to account for sensorimotor delays in the central nervous system (Nijhawan, 1994). Furthermore, the occlusion of visual information would result in a considerable impairment of the action. A possible solution to provide adequate responses would be to continue to do what has been done so far (Bootsma, 2009). Nevertheless, in temporally constrained tasks, another question arises: for how long? In the outfielder problem, catching the ball in-flight imposes tight temporal restrictions constraining the interception area. Hence, predicting parameters such as TTC or the interception area in dynamic contexts is key to planning our actions while looking around for a teammate, finding a safe path towards the goal, or modulating speed to reach the interception location in time. Following this reasoning, some alternatives have been proposed in the literature as solutions based on predictions about future states of the world.

### Model-Based Control

The model-based control is framed within the constructivist framework (Von Helmholtz, 1867). It assumes that the information picked up by our senses is inherently ambiguous, to some degree corrupted by noise in the neural system and delayed at higher-order brain areas. Craik (1967) proposed that the brain tries to infer and replicate an external world model given the available sensory information. This replica results in an internal model of the environment, including an agent’s state that allows one to predict future states of the world and act accordingly avoiding sensorimotor delays.

Relying on predictions would allow us to divert our gaze from the immediate region of interest and consequently interrupt the sensory flow. For example, Hayhoe et al. (2005) and Diaz et al. (2013) showed anticipatory saccades towards future interest points to plan future goal states. The same applies to catching a ball in a parabolic trajectory for manual interception tasks. Despite distractors, parallel tasks, occlusions or head-turns that might divert our attention, we still manage to intercept a ball in flight (Dessing et al., 2009; López-Moliner et al., 2010; López-Moliner and Brenner, 2016; Binaee and Diaz, 2019) In fact, we can hit it even when the ball was visible for just a short time (Sharp and Whiting, 1974; Whiting and Sharp, 1974; Amazeen et al., 1999), revealing that actually, the major constraint for the use of a predictive strategy would be to obtain predictions early enough to overcome sensory-motor delays.

The trajectory prediction strategy (Saxberg, 1987a,b), framed within the model-based theory, assumes that an observer predicts where and when the ball will be within reach in Cartesian units, allowing to pre-program a minimal action plan since motion onset. However, the available optic information is egocentric and therefore ambiguous with respect to its source. Therefore, an observer must perform an inferential process to interpret optic information accurately.

In this line, Perception has been proposed as a Bayesian inferential process in which visual information is interpreted as a function of the most probable state of the world given prior knowledge. This inferential process has been formulated in terms of “encoding” and “decoding” (Knill and Pouget, 2004; Friston, 2010; Wei and Stocker, 2015). Encoding corresponds to the activity resulting from the transduction of external energy onto the sensory receptors. The encoded sensory information is then combined with prior knowledge through an inferential process called decoding (Figure 1B). The product of the decoding is an interpretation (read-out) of the currently available data resulting in a belief of the state of the world that provides the grounds to draw predictions.

In real life, we generally do not judge the parameters of a task for ambiguous targets in the environment. Instead, we have some prior knowledge of the elements to be judged that are stable and might help disambiguate optic information providing the grounds to extract valuable information for the task. In this line, a significant number of works highlighted the role of prior knowledge about contextual variables such as gravitational acceleration (McIntyre et al., 2001; Jörges and López-Moliner, 2017) or known/familiar size (López-Moliner et al., 2007; Hosking and Crassini, 2010), framing the interpretation of visual information for the control of timed actions.

Note that using *a priori* knowledge does not imply the availability of accurate Cartesian metrics or Newtonian laws within an internal model. A fully-featured 3D internal model replicating the external world has been repeatedly dismissed. For example, Shaffer and McBeath (2005) showed that even expert baseball players could not judge the apex of a ballistic trajectory on a collision course with the observer. In this situation, the apex was estimated to be 0.33 s before collision for flight durations of 4 s, that is, 1.66 s after the actual apex. These results indicate a tendency to judge the apex of the elevation angle as the physical apex of the trajectory. Also, Reed et al. (2010) showed that neither expert nor novel baseball players could reconstruct the visual trajectory of a parabolic trajectory in a head-on approach mixing up the ball’s movement in space with the visual trajectory it follows.

Unlike Craik (1967), however, we propose that the prior knowledge can be kept to a minimal number of components that help exploit the optic flow’s complexity. Thus, in line with a Bayesian framework, under our view, the use of prior knowledge just suggests the existence of a probabilistic and implicit knowledge acquired by repeated experience that helps infer the most probable sources of visual information in the external world (Zago et al., 2008; Gómez and López-Moliner, 2013). In this sense, an accurate representation of *a priori* parameters would suffice to obtain reliable estimates of the task’s parameters.

Here we propose using priors as internalized knowledge to translate optic variables into temporal estimates in a process we name calibration. Calibration would be the process by which optical or angular information is mapped into Cartesian ones with the assistance of different pieces of prior knowledge providing actionable predictions (López-Moliner et al., 2007, 2013). Calibrating optic cues into Cartesian allows us to test the correspondence between the prediction of a model and our actions in terms of accuracy. Furthermore, it may allow us to formulate a hypothesis based on known psychophysical precision levels for the different pieces of information in the sensory array and even check if integration rules apply (Wolpert et al., 1995; de la Malla and López-Moliner, 2015).

As an example, the GS model (Gómez and López-Moliner, 2013) is an algorithm that predicts the TTC for parabolic trajectories based on a combination of optic variables and prior knowledge information. Its predictions have been partially validated based on predictions about the accuracy and the precision of temporal estimations (de la Malla and López-Moliner, 2015; Aguado and López-Moliner, 2021). However, there is a lack of mathematical formulation to predict the interception location. This makes it harder to test experimentally predictive control strategies within the outfielder problem. Hence, in this work, we will limit ourselves to indicate the role of TTC estimates guiding the interceptive action.

It is essential to mention here that our definition of calibration is different from the definition of calibration made within the Ecologic framework (Fajen, 2005a; Jacobs and Michaels, 2007). Under our perspective, calibration is a process by which otherwise ambiguous optical information is directly mapped into kinematic and temporal estimates such as motion vectors or TTC highlighting the relevance of prior knowledge to provide predictions that may assist visually guided actions. In contrast, within the Ecological framework, calibration would be a by-product of a gauging process that maps visual information into movement information and even into optic correlates in object size units (Peper et al., 1994; Jacobs and Michaels, 2006).

Nevertheless, producing predictions does not necessarily mean that those predictions will be accurate or that the new visual information would be disregarded. Take the case of Fink et al. (2009) study. Participants had to catch a ball in a parabolic trajectory that suddenly would alter its motion towards the ground. As a reaction, the catchers changed their trajectory towards the ball as well which was taken as support for the information-based control perspective. Under Fink et al. (2009) rationale, a model-based control strategy would result in a consistent path towards the interception point despite mid-flight disturbances. This rationale assumes that new information would be dismissed or might be irrelevant because the prediction would remain invariant. However, predictions would also be subject to continuous evaluations to avoid errors or perceptually driven biases. In a similar line, Postma et al. (2014) reasoned that continuously gazing the ball through the trajectory would support the information-based control perspective. However, following the ball with our gaze does not necessarily imply that action guidance must be driven by instantaneous optic information. Periodically sampling visual input to correct the prediction made would be an alternative strategy to guide action with reasonable levels of accuracy in a more general framework (Brenner and Smeets, 2018). In fact, a simulation study conducted by Belousov et al. (2016) showed that predictive behavior would be indistinguishable from using error-nulling strategies if the ball is continuously monitored.

In the following sections, we will unveil how the interaction between perceptual information and prior knowledge contributes to interpreting and reliably predict TTC for gravitationally accelerated objects under parabolic trajectories. To do so, we will first analyze the available sources of visual information within the optic flow to judge TTC or time an interceptive action. Then, we will stress the role of prior information in calibrating visual information. After that, we will show the accuracy and reliability of the GS model which includes known gravity and size in complex environments. Finally, we will indicate future lines of research to address the role of predictions of TTC guiding interceptive behavior.

## Available Visual Information

When an observer faces a ball in a parabolic trajectory, the projectile describes the following sequence of events (see Figure 2). The ball initially goes up at a decreasing speed until it reaches the peak of its trajectory. Then, it accelerates during the descent towards the ground. However, an observer cannot access the underlying dynamics of projectile motion using Cartesian metrics. Instead, they only have access to information based on egocentric angular variables that depend on their position and the kinematics of the ball (Shaffer and McBeath, 2005; McBeath et al., 2018). Hence, the ball’s kinematics and an observer’s movement influence the visual information being exposed throughout the trajectory. Figure 2 depicts the main primitive optic variables that we will consider and how both unfold over time (*t*) depending on the observer’s position and total flight time (*T*), which is unknown to the observer.

While interpreting sensory information is a central part of this work, we also need to consider the limitations of our sensory system to gather that visual information. Because of that, in the following subsections, we will elaborate on the conditions that render different visual cues helpful regarding detectability or discriminability, describing their precision as Weber fractions. To do so, we will assume that the observers keep their gaze on the projectile, which is usually the case in free viewing situations (Oudejans et al., 1999; Postma et al., 2014) and laboratory-controlled tasks when the position to hit a target is not pre-specified (Brenner and Smeets, 2007; Soechting et al., 2009; Cámara et al., 2018).

### Retinal Size (*θ*)

The visual angle or retinal size is the angular size projected by an object on the retina (*θ*; see green projections in Figure 2). Retinal size (*θ*) is proportional to both object size and distance, being the prototypical example of an ambiguous optic variable. Previous studies have shown that human performance in discrimination tasks for angular size judgments is about a 3–6% Weber fraction (WF; *k*_θ_) for objects yielding >0.0009 radians, increasing steeply up to a 20–30% (WF) for smaller objects (Westheimer and McKee, 1977; Klein and Levi, 1987; McKee and Welch, 1992).

Retinal size is a zero-order variable; that is, it does not carry temporal information and thus, cannot be employed alone to estimate motion components or TTC. To do so, one needs to have access to the rate of expansion ($\dot{\delta }$), which is the speed at which the retinal image changes. The absolute detection threshold for $\dot{\delta }$ has been reported to be about 0.0003 rad/s (McKee, 1981), while the discrimination threshold associated with this parameter is about an 8.5–14% of change (WF; *k*_$\dot{\delta }$_; Regan and Hamstra, 1993).

In some cases, such as baseball games, the players meet scenarios where the ball is at a considerable distance. In those cases, although mediated by physical size and ball’s horizontal speed, discriminability of the rate of expansion ($\dot{\delta }$) is generally poor (20% of change (WF) for objects expanding at a rate of less than 0.004 rad/s (Regan and Beverley, 1978; Harris and Watamaniuk, 1995).

**Figure 3:**
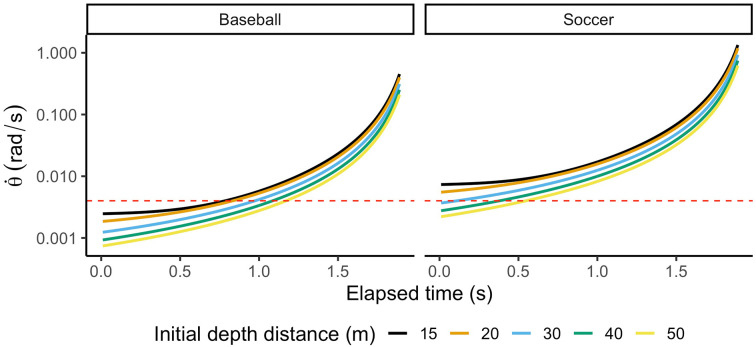
Rate of expansion ($\dot{\delta }$) for two different ball sizes at five different initial distances. The total flight time is 2 s. Values under the red dashed line (0.004 rad/s) indicate that an observer cannot discriminate differences.

To show the effect of ball size on an observer’s ability to discriminate differences in the rate of expansion ($\dot{\delta }$), we computed the rate of expansion for two different balls (baseball and soccer balls) moving in a parabolic trajectory towards an observer from different initial distances. Figure 3 shows how retinal expansion unfolds as a function of time. Values below 0.004 rad/s (red dashed line) would fall below the optimal discriminability range, indicating that the observer’s ability to discriminate differences is inferior. As depicted in Figure 3, a player facing a baseball will not discriminate retinal expansion during more than half of the trajectory. Instead, differences in retinal expansion can be discriminated during most of the trajectory of the Soccer ball. This example aims to point out that even when visual cues are present in the optic flow, the resolution of our visual system might not allow us to use them to guide our actions. Therefore, initial estimates of TTC might be computed using alternative routes.

### Tau (τ)

Lee’s seminal work (Lee, 1976) described Tau (from now on, *τ*) as the ratio between the visual angle (*θ*) and its rate of expansion ($\dot{\delta }$; for a review, see Hecht and Savelsbergh, 2004). Tau signals TTC and is directly accessible within the optic flow without prior knowledge or previous estimates of distance, size, or approaching speed.

Although *τ* can not be conceptualized as a primitive variable in the study of visual cues, it has shown different features that could allow us to consider it as such. Regan and Hamstra (1993) found that differences in *τ* could be distinguished independently of differences in *θ* or $\dot{\delta }$ (*k*_τ_ ≈ 0.07:0.13; WF). Because of this, Regan and Hamstra (1993) concluded that there might exist a mechanism sensitive to *τ* independently of *θ* or $\dot{\delta }$. Indeed, other studies have shown the existence of a neural mechanism tuned to *τ* (independently of retinal size and rate of expansion) or some of its modifications [such as the *η*-function (Judge and Rind, 1997) or *τ*_m-function (Keil and López-Moliner, 2012)] in various species such as pigeons and humans (Yonas et al., 1977; Sun and Frost, 1998; Rind and Simmons, 1999).

Tau (*τ*) has been indicated as a source of prospective information that might be used as a threshold (as a criterion) to perform different tasks such as hitting (Lee et al., 1983; Bootsma and van Wieringen, 1990) or catching (Savelsbergh et al., 1991). This threshold is usually referred to as Tau-margin (Wann, 1996). However, its applicability to time parabolic trajectories might be compromised due to several limitations.

First, *τ* would only generate accurate TTC predictions at launch when the ball travels in a collision course with the observer.

Second, the object should approach the observer at a constant speed. In a parabolic trajectory, from an allocentric viewpoint, *V*_x_ and *V*_z_ are constant (assuming no air resistance). Nevertheless, the approaching speed for the observer corresponds to radial velocity (*V*_r_), which would carry the isotropic expansion of the retinal expansion. However, *V*_r_ is not constant through the trajectory and cannot be directly estimated from the optic flow (Gómez and López-Moliner, 2013). For instance, consider a trajectory launched 2 m far from an observer in a trajectory 10 m of height. In this situation, during the first half of the trajectory, *V*_r_ and retinal expansion $\dot{\delta }$ would be negative; that is, the object moves further away from the observer, rendering meaningless estimates of TTC using Tau.

Third, even though Tau could be discriminated independently of the rate of expansion ($\dot{\delta }$), it is likely constrained to the same detection thresholds (Keil and López-Moliner, 2012). Therefore, rates of expansion ($\dot{\delta }$) lower than 0.004 rad/s could result in a non-informative source to guide temporal estimations during an important section of parabolic trajectories. As reported above, the WF for Tau ranges between 7% and 13%. Therefore, we will be using a mean WF of 10% referring to Tau in the following sections.

Finally, it might not be directly implemented as a general-purpose mechanism because the object must be spherical and rigid, which is not the case for an interception in some sports such as Rugby or Frisbee.

### Elevation Angle (γ)

The elevation angle (γ) is the position of an object in the vertical meridian of the retina (see orange projection in Figure 2. We conceptualize the elevation angle (γ) using a spherical projection because angular variables are assumed to be directly accessible to an observer (McBeath et al., 2018).

Even though it is often referred to as the vertical position of an object in the retina, when a projectile is visible, we tend to perform continuous visual follow-ups foveating the object, which might prevent perceptual biases (de la Malla et al., 2017). In those cases, the retinal angle of the elevation angle (*γ*) would tend to zero. Because of that, it has been suggested that this visual angle can be estimated as a combination of the displacement of the environment in the retina (Oudejans et al., 1999; Brenner and Smeets, 2015b), the movement of the eyes with respect to the observation axis (Crowell and Banks, 1996) and estimates of the heading angle generated at the otoliths of the vestibular system (Berthoz, 2000; Roy and Cullen, 2003) produced by the movement of the head and trunk (Crowell et al., 1998; Lewis et al., 1998). Previous literature has found that foveated objects require a difference of up to a 3–5% of change (WF) to be effectively discriminated (Regan and Kaushal, 1994; Crowell and Banks, 1996).

The first derivative with the time of the rate of change of the elevation angle ($\dot{\gamma }$) is the vertical rate of displacement of a target in the retina (see Figure 2). According to several studies (McKee, 1981; Orban et al., 1984; de Bruyn and Orban, 1988), the ability to judge differences in $\dot{\gamma }$ is about 5% (WF) for angular velocities between 0.03 rad/s (1.71 deg/s) and 1.2 rad/s (69 deg/s). Interestingly, Portfors-Yeomans and Regan (1996) suggest channels that process position and cardinal motion independently, which indicates that the noise for both estimates is independent.

Given that parabolic trajectories move accelerated by terrestrial gravity, it is reasonable to consider humans’ ability to detect acceleration. Calderone and Kaiser (1989) proposed that acceleration in the visual system can be studied as the rate of change in speed divided by the average object speed in a two-stage process carried out in about 200 ms (Werkhoven et al., 1992; Zaal et al., 2012). This delay would mean that the observer would not continuously monitor the adequacy of their actions. Furthermore, some studies found that it is necessary at least ~20% of the change in speed to detect acceleration (Gottsdanker et al., 1961; Werkhoven et al., 1992; Babler and Dannemiller, 1993; Brouwer et al., 2006; Zaal et al., 2012) indicating that humans are quite insensitive to changes in speed.

### Disparity (δ)

Disparity (δ) is the vertical/horizontal difference between the position of an object in the retinal image of both eyes. An algorithm like Tau (τ) for the estimation of TTC has been proposed as a combination of the knowledge of interocular distance (I), the distance with the ball (D) and the rate of change of horizontal disparity) ($\dot{\theta }$) (TTC ≈ I/(D*$\dot{\theta }$)). Furthermore, it can be used to estimate the lateral distance at which an object would pass an observer position.

A combination of Tau and the information contained in the above expression would assist the estimation of TTC to achieve our exceptional temporal precision batting fastballs (Gray and Regan, 1998). This solution may account for systematic underestimations of TTC by weighting the visual cue that indicates a shorter TTC to guide the final interceptive phase (Savelsbergh and Whiting, 1992; Gray and Regan, 1998; Rushton and Wann, 1999). However, Brenner et al. (2014) found no evidence that hitting a free fall ball uses the rate of change in disparity ($\dot{\theta }$) to estimate TTC. In this sense, Brenner and Smeets (2018) argue that some studies that compare the performance between monocular and binocular conditions “ignore the benefit of having two estimates of the relevant monocular cues” instead of one.

## Evidence of Prior Knowledge Calibrating Visual Information

Humans quickly acquire knowledge about regularities in their interaction with the environment. Regularities such as the light coming from above (Adams et al., 2004), that bigger means heavier (Peters et al., 2016) or the fact that object size is generally constant (López-Moliner and Keil, 2012) enhance predictability and reduce uncertainty about future states of the world. Indeed, the assumption of a stable world is at the heart of essential findings in different areas of vision science, such as speed perception (Stocker and Simoncelli, 2006), depth estimation (Glennerster et al., 2006), and manual interception (Brenner and Smeets, 2015a).

Such known regularities may also be referred to here as contextual information stressing the role of acquired knowledge by repeated experience within a specific context. In this work, we will focus on two pieces of internalized knowledge that usually remain stable in our world and might frame the interpretation of visual information for parabolic trajectories: known size and gravity.

### Size

The assumption of constant size is likely one of the most critical assumptions about action because, in general, the objects around us do not change size unexpectedly. Under this assumption, known size calibrates visual information into distance estimations with an object as the ratio between known size and the retinal size projected (Ittelson, 1951; Hecht et al., 1996; Sousa et al., 2011) even though irregular objects such as rugby balls and frisbees can be problematic.

(1) $$d=\frac{s}{\theta }$$

The previous expression provides a mechanism to scale the optic space into ball size units (Peper et al., 1994; Gómez and López-Moliner, 2013) for a broad range of contexts. Calibrating the optic space with known size provides estimates of relative distances in paintings, pictures, video games, and environments with low or incongruent pictorial detail (Todd, 1981; Saxberg, 1987b), sometimes at the cost of leading to systematic misperceptions (Battaglia et al., 2005; Tcheang et al., 2005; Battaglia et al., 2011).

In line with the use of known size calibrating visual information, López-Moliner et al. (2007) showed that when an object approaches at a constant speed and physical size is known; an observer can exploit the lawful relations between physical size and optic variables in the equation above to estimate approaching speed (*V*_z_) as its first derivative:

(2) $$V_{z}=\frac{s\dot{\theta }}{\theta^{2}}$$

In the above expression, known size (*s*) allows calibrating retinal size (*θ*) and rate of expansion ($\dot{\delta }$) into an estimate of approaching speed (*V*_z_) from otherwise spatiotemporally ambiguous optic variables. Then, an observer could use single optic variables (e.g., retinal size or expansion rate) to time the initiation of interceptive actions (Smith et al., 2001; López-Moliner et al., 2007; López-Moliner and Keil, 2012) depending on noise levels (Aguilar-Lleyda et al., 2018). However, what happens when it comes to obtaining parameters of more complex tasks such as parabolic trajectories?

In Todd (1981), the participants had to judge if a ball on a parabolic trajectory would fall in front or behind an observer in different experimental conditions. Todd’s work showed that, even though there might be enough information to estimate the final position qualitatively for parabolic trajectories only based on sensory information, prior knowledge of an object’s size helped the participants to judge the final position in depth accurately. In each block, the absolute size was either fixed, selected at random, or fixed to a single dot during the whole trajectory. Accuracy was significantly better when the absolute size was fixed than the condition in which size was selected randomly. Those results indicate that prior knowledge of the ball’s size aids the estimation of motion-in-depth.

Interestingly, the third condition yielded the worst performance of all three conditions, yet performance was slightly over chance level. In this condition, only the vertical movement was available to the observers to judge approaching speed. Participants judged landing position based on “the amount of vertical speed.” In line with these results, Jörges and López-Moliner (2017) showed that prior knowledge of gravity might be essential to calibrate estimates of the rate of change ($\dot{\gamma }$) into estimates of approaching speed (*V*_z_) in parabolic motion.

### Gravity

Since Lacquaniti and Maioli (1989) work, showing an anticipatory activity for gravitationally accelerated objects, there is evidence that an internal representation of gravity may play a key role in controlling interceptive actions and judging TTC. For example, McIntyre et al. (2001) found that astronauts react to moving objects as if they were accelerated by Earth gravity under micro-gravity conditions. That study showed that although the astronauts were immersed in an environment where visual and bodily cues indicated microgravity conditions, they could not adapt their interceptive actions completely. After 15 days, the astronauts were still anticipating their interceptive actions mimicking the conditions under terrestrial gravity. Subsequent work using virtual reality setups showed that participants could adapt to arbitrary gravities in a few trials. However, the performance is still lower than that under terrestrial gravity conditions (Zago and Lacquaniti, 2005; Zago et al., 2005).

Since then, an implicit representation of gravity has been found at a neurobiological level (Indovina et al., 2005; Miller et al., 2008) and in a broad range of tasks such as eye behavior (Bosco et al., 2012; Diaz et al., 2013; Jörges and López-Moliner, 2019) or the estimation of the duration of events (Hosking and Crassini, 2010; Moscatelli and Lacquaniti, 2011; Jörges and López-Moliner, 2020) despite our general insensibility to accelerations (Werkhoven et al., 1992).

However, the best example of a representation of gravity for sensorimotor control is that an observer does not need to see an ascending ball falling to intercept it (de la Malla and López-Moliner, 2015). In general, humans have an implicit expectation that upwards moving objects will eventually fall (López-Moliner et al., 2010; Reed et al., 2010). Nevertheless, this representation may not be available for every kind of task. For example, timing tasks for gravitationally accelerated objects in imagination show a bias towards the last visible motion speed (Gravano et al., 2017; Bratzke and Ulrich, 2021). In this line, some authors dismiss an internal model-based explanation favoring a prediction-free explanation (Baurès et al., 2007; Katsumata and Russell, 2012). However, the lack of adaptation under microgravity conditions and the need to account for sensorimotor delays pinpoint the relevance of gravity prior guiding predictive control (Zago et al., 2008).

A recent study (Jörges and López-Moliner, 2020) tried to derive the mean and standard deviation of the Gravity prior in a Bayesian framework. Their results found a prior with a standard deviation of 14% (WF). According to the authors, these results might correspond with an upper bound, as there seem to be theoretical reasons such as the lack of adaptation to arbitrary gravity values suggesting a relatively inflexible and robust gravity prior (Jörges and López-Moliner, 2017).

To test the use of different pieces of prior information for calibration, it is first necessary to put forward algorithms that require pieces of internalized knowledge. In the temporal domain, Gómez and López-Moliner (2013) showed that by using both prior knowledge of gravity and size, visual information could be calibrated, resulting in actionable estimates of TTC. The model was named GS model about the assumption of *a priori* known gravity and size.

## Time-To-Contact Estimation

### GS Model

The GS model (Gómez and López-Moliner, 2013) is an algorithm that relies on calibrated optic information using prior knowledge to obtain estimates of TTC for parabolic trajectories. It relies on a combination of contextual variables such as known ball size (*s*) and gravitational acceleration (*g*) along with monocular cues such as retinal size (*θ*), elevation angle (*γ*) and its first derivative ($\dot{\gamma }$), providing accurate estimates of TTC.

(3) $$TTC_{GS}\approx \frac{2}{g}\frac{s}{\theta }\frac{\dot{\gamma }}{\cos\;(\gamma )}$$

Known ball size (*s*) and retinal size (*θ*) provide a mapping from retinal to Cartesian metrics. On its part, gravitational acceleration (*g*) calibrates and normalizes the rate of change of the elevation angle ($\dot{\gamma }$) to be interpreted into meaningful predictions of TTC under arbitrary gravitational accelerations. In addition, cos(γ) would act as a non-declarative internalized parameter linked to action expecting that the elevation angle (*γ*) would increase over time (Reed et al., 2010; Shaffer et al., 2013). Removing the internalized variables from the GS model, one can still obtain a correlate of TTC based on retinal size (*θ*), the elevation angle (*γ*) and the rate of change of the elevation angle ($\dot{\gamma }$). However, its value is meaningless in signaling an actionable TTC and, therefore, not directly applicable. The following section will illustrate that envisioning those pieces of prior knowledge as priors within an encoding-decoding framework could calibrate ambiguous visual cues into accurate estimates of TTC for parabolic trajectories.

#### Simulating the Benefits of Using Gravity and Size Priors for the Decoding

Under the constructivist framework, visual information is underspecified, and many trajectories can originate the same stimulation. This simulation shows how entering the correct gravity and size values increases the chances of inferring the actual trajectory exposed to the system from a subset of the possible ones.

In the case of this simulation, the possible inferred parabolas are a combination of the nine different TTC (ranging from 1.8 to 2.2 s in steps of 0.05 s), five different conditions of gravity (8.826, 9.316, 9.807, 10.297, 10.787 m/s^2^) and five different sizes (0.0703, 0.07215, 0.074, 0.07585, 0.0777 m) launched at eye-level 30 meters away from the observer in a head-on approach. We chose those values of gravitational acceleration and size using differences of 5% the standard of Earth gravity and 2.5% of the standard size of a baseball to envision reliable values of prior knowledge of gravity and size (however, see Jörges and López-Moliner, 2020).

**Figure 4:**
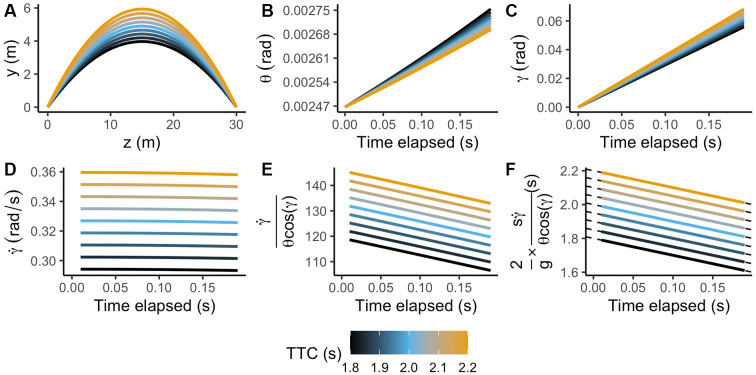
Test parabolas used in the simulation. **(A)** Ball’s vertical vs. depth position. Panels **(B–D)** indicate retinal size (*θ*), elevation angle (*γ*) and rate of change of the elevation angle ($\dot{\gamma }$) as a function of time for the first 200 ms of the trajectory. Panels **(E,F)** depict the output of the GS model using visual information only and combined with prior information, respectively.

Figure 4 represents nine test trajectories exposed to the system (panel A), whereas panels B, C and D represent the range of possible values for each variable available for the observer: retinal size (*θ*), the elevation angle (*γ*) and the rate of change of the elevation angle ($\dot{\gamma }$) respectively.

**Figure 5:**
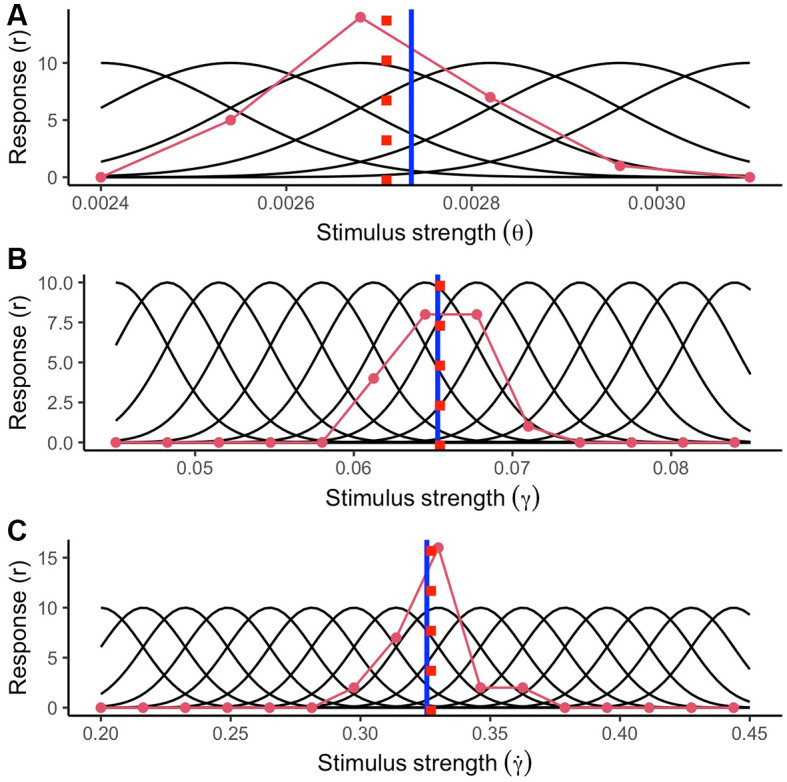
Simulated tuning curves of neurons specialized for different values of **(A)** retinal size (*θ*), **(B)** elevation angle (*γ*) and **(C)** rate of change of the elevation angle ($\dot{\gamma }$). The blue vertical line indicates the true stimulus strength exposed to the system. The stimulus strength was selected from the standard condition 200 ms after motion onset (see main text). The red curve indicates the average activation per neuron in a single trial (Poisson noise added). The red dashed lines indicate the stimulus strength inferred by the encoding process.

To reproduce the encoding process of a sensory stimulus, we simulated a set of tuning curves covering the range of possible stimulus strengths (stimulus values) for each optic variable 200 ms. after motion onset. In Figure 5, the reader can see an illustration of the tuning curves (black curves), the stimulus strength presented to the system (blue vertical line), and an example of the average response by a detector (red curve) for the average TTC under standard conditions of gravity and size (light blue trajectory in Figure 4). The detectors simulated for retinal size (*θ*) covered a range from 0.0024 to 0.0031 rad (SD = 0.00014; representing a 5% WF). The stimulus range covered for the elevation angle (*γ*) and its rate of change ($\dot{\gamma }$) was from 0.045 to 0.085 rad (SD = 0.00325 rad; 5% WF) and from 0.2 to 0.45 rad/s (SD = 0.01625 rad/s; 5% WF) respectively.

After the detectors were exposed to the stimulus, we obtained the average response probability (r; solid red line in Figure 5) for corresponding neural detectors (Dayan and Abbott, 2001) as:

(4) $$p\;(r)=f{\left(\theta ,\gamma ,\dot{\gamma }\right)}^{r}\frac{e^{-f(\theta ,\gamma ,\dot{\gamma })}}{r!}$$

In this expression, (*f*) indicates the mean activity per detector, whereas (*r*!) corresponds to the factorial response for each detector. In our simulation, the resulting activation varies on each iteration by adding Poisson noise, representing random variability in neural activation.

We simulated 1,000 trials per TTC in which the size was the standard of a baseball (0.074 m), and gravity was the standard on Earth (9.807 m/s^2^). Once the optic variables were encoded by the detectors simulated, we recovered the most likely stimulus strength presented to the system for each optic variable using a Maximum Likelihood estimate (MLE) procedure. Then, we obtained a value corresponding to the GS model based only on the optic variables retrieved by the encoding procedure.

We compared that output to the GS model’s ideal (noiseless) output based only on optic variables for all the simulated trajectories. We obtained two possible sets of decoding responses to select all the potential trajectories that matched the model’s output. For the first set of responses, we only used sensory information without resorting to size or gravity priors to decode the correct TTC; that is, we used a Maximum Likelihood Estimation procedure. We assumed the correct size and gravity priors to decode the correct TTC for the second set of responses. Then, we selected all potential trajectories that would fall into a relatively low error margin of ± 5%.

**Figure 6:**
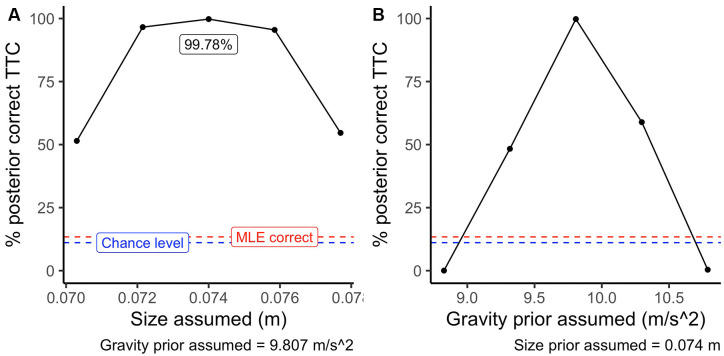
Average accuracy per procedure. The blue dashed line indicates chance level (11.1%), red dashed line indicates the performance of an maximum likelihood estimate (MLE) procedure. The black points indicate the performance assuming different size priors in panel **(A)** (*g* = 9.807 *m/s*^2^ assumed) and assuming different gravity priors in panel **(B)** (baseball size assumed).

Figure 6 depicts the average accuracy per procedure. Here, the performance of a system using only sensory information (MLE; red dashed line) is slightly over the chance level (blue dashed line). On the contrary, using the correct priors (central dots in panels A and B of Figure 6) improves the proportion of correctly estimated TTC’s substantially. Note that since the relative difference between the different simulated gravity values is higher than those of size, the procedure benefits more from an accurate representation of internalized knowledge of gravity. This example highlights that, despite the inherent ambiguity of sensory information in the optic flow, the use of prior information is a powerful calibration tool to interpret otherwise ambiguous visual information.

### Accuracy and Precision of Using Gravity and Known Size

So far, we have shown that calibrating visual information in the light of prior knowledge allows us to draw accurate predictions using the GS model. However, it could still be the case that even if the output is accurate, the visual information in the optic flow is so noisy that an estimation of TTC might not be available.

To evaluate an observer’s ability to estimate TTC accurately and precisely from the GS model, we simulated a series of typical spatio-temporal parameters for parabolic trajectories. We simulated parabolic trajectories launching at eye-level at five initial distances (*Z*_init_ = 15, 20, 30, 40, 50 m), one contact time (*TTC* = 2 s; Δ_t_ = 0.01 s) and a single radius corresponding to a baseball (0.037 m). In each case, the endpoint is the origin, that is, the position of the simulated observer.

To evaluate the precision of the output, we introduced independent Gaussian noise to *θ*, *γ* and $\dot{\gamma }$ according to their respective WFs (identified with the letter *k*).

(5) $$\theta_{\chi }=\theta +N_{\left(\mu =0;\;\sigma =\theta *\theta_{k}\right)}\;\theta_{k}=0.05$$

(6) $$\gamma_{\chi }=\gamma +N_{\left(\mu =0;\;\sigma =\gamma *\gamma_{k}\right)}\;\gamma_{k}=0.05$$

(7) $${\dot{\gamma }}_{\chi }=\dot{\gamma }+N_{\left(\mu =0;\;\sigma =\dot{\gamma }*{\dot{\gamma }}_{k}\right)}\;{\dot{\gamma }}_{k}=0.05$$

To test the noise-suppression performance for the GS model, we ran 10,000 simulations for each condition using Equations (5)–(7). Then, we obtained a WF timewise as the ratio between the standard deviation of the signal and the mean predicted TTC. Note that the χ version of a variable denotes its noisified version.

**Figure 7:**
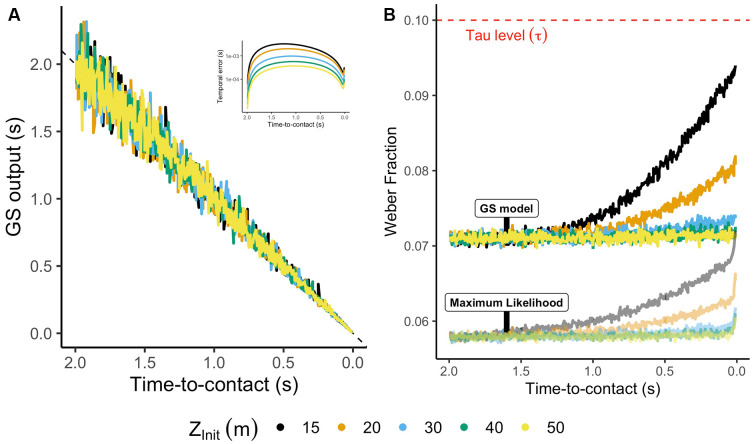
**(A)** Noisified estimates of TTC using the GS model for different trajectories. The inset represents the temporal error for the noiseless output of the GS model. **(B)** Weber fraction (WF) computed as the ratio between standard deviation and mean of the GS model each temporal frame. The red dashed line indicates the mean WF of Tau (see main text). The translucid output indicates the WF of a combination of the GS model and Tau using an MLE procedure.

Figure 7A depicts the output of the GS model for each initial distance (color code), whereas the inset indicates the predicted temporal error for the ideal (noise-free) output of the model. The GS model provides very accurate estimates for which the maximum error is lower than 10 ms. On its part, Figure 7B represents the WF estimated timewise for the GS model. As a comparison, the WF for Tau was envisioned as constant at 10% as reviewed above. The GS model presents a precise output during most of the trajectory (WF is always lower than 10%), resulting in an accurate and robust solution to the estimation of TTC in parabolic trajectories comparable to previous WFs found in the literature (Moscatelli and Lacquaniti, 2011; Jörges and López-Moliner, 2020).

It is essential to mention here that just as others have already described in the literature, the sources of information to estimate TTC may vary depending on the segments of an approach visible (DeLucia, 2004; López-Moliner et al., 2013; DeLucia et al., 2016). While an initial temporal estimate would be available using the GS model, final interceptive actions would take advantage of more straightforward strategies such as a distance criterion (Wann, 1996; López-Moliner and Keil, 2012; Gómez and López-Moliner, 2013), Tau (Lee, 1976; Zago et al., 2004; Shaffer and McBeath, 2005) or correlates of binocular disparity (Rushton and Wann, 1999).

Following this reasoning, de la Malla and López-Moliner (2015) partially validated the use of the GS model, showing that early estimates of TTC based on the GS model could be integrated with the latest estimates derived from correlates of the rate of expansion resulting in an accurate and precise timing mechanism. Mimicking that context, we combined the predictions of Tau and the GS model using a maximum likelihood process (Ernst and Banks, 2002). The output results in a robust solution against sensory noise for the estimation of TTC and timing interceptive actions (see translucid lines in Figure 7B).

### Generalization of the GS Model

The formalization of the GS model assumes that the ball moves in a collision course with the observer (Gómez and López-Moliner, 2013). However, this is not usually the case. Commonly an observer must move to intercept the ball. Therefore, the following question is to what extent the output of the GS model deviates from perfect accuracy for trajectories ending in locations other than the observer’s position?

**Figure 8:**
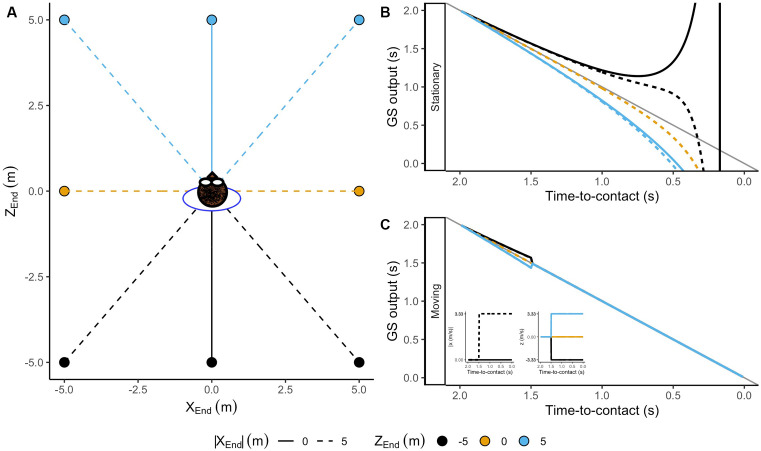
**(A)** Ending positions for simulated trajectories around the observer. The lines represent the trajectories followed by the moving observer. Panels **(B,C)** depict the output of the GS model for a stationary and a moving observer. The line code indicates lateral ending position [*X*_End_ = 0, 5 (m)]. The color code indicates the ending position in depth [*Z*_End_ = −5, 0, 5 (m)] . Note that the GS model predicts an underestimation of TTC for balls falling ahead and an overestimation of TTC for those falling behind the observer.

To investigate those cases, we estimated the output of the GS model for trajectories ending at different interception locations. We simulated one initial distance (*Z*_init_ = 50 m) and eight interception points around the observer (see Figure 8A). Initially, the GS model provides accurate estimates of the TTC regardless of the position of the observer. Then, in contrast with trajectories on a collision course, the simulation reflects systematic errors in TTC estimation shortly after motion onset if the observer remains stationary (see Figure 8B). If the ball falls behind the observer, the rate of change of the predicted TTC decreases. Thus, the model’s output overestimates the remaining TTC and *vice versa* for balls falling ahead (see Figure 8B), pointing out that the errors depend on the interception location. In this context, a navigational strategy predicting where and when the ball would be within reach would initially guide the observer towards the wrong position.

Nevertheless, the simulations described so far result in predictions of TTC for an unlikely situation in which the observer is not at the interception location and remains stationary. Usually, an observer would control the ball moving towards the interception area. As a result, an observer’s movement would prompt changes in the optic flow. As we will show, simulating the observer’s movement, we found interesting properties in the output of the GS model that might indicate the availability of a navigational strategy.

To perform the following simulations, we replicated the previous trajectories. However, in this case, the observer started moving towards the interception location at a constant speed 500 ms after the ball’s launch. We chose this moment because catchers generally start running in the right direction 500 ms after the ball’s flight has started (Michaels and Oudejans, 1992; McLeod and Dienes, 1993, 1996; Hecht et al., 1996; Brouwer et al., 2006). The displacement speed of the observer was computed to reach the interception point just in time to catch the ball (see inset in Figure 8C). Although this pattern of displacement and speed does not correspond precisely to the found in real life (McLeod and Dienes, 1993; McLeod et al., 2006), it is essential to point out that it will be useful for an illustrative purpose.

In Figure 8B, the reader can see that when the observer remains stationary for some time in a position other than the interception location, the rate of change of the predicted TTC changes (Figure 8B). When the rate of change in TTC decreases, the ball will fall behind the observer and *vice versa*. This would signal the need to move and also provide information of the correct direction of movement in depth. Therefore, departures from the initial rate of change in TTC (slopes different from −1) could be used as a navigational strategy indicating if the observer must move forward or backwards. Instead, the movement of an observer in the correct direction provides the necessary change in the optic flow to linearize the predictions of the remaining TTC (see Figure 8C). Thus, keeping the prediction linear will ensure that the observer would end up at the interception position in time.

In sum, the above simulations indicate that the model’s output is accurate when the observer moves in the correct direction and speed providing the basis for a mechanism to navigate towards the interception location. However, these simulations were performed in a context in which the ball is only affected by the gravitational acceleration. Would a simulation of trajectories under air drag provide equally accurate temporal estimates?

### Dynamic Effects: Air Drag

In real life, the ball is affected by external forces other than gravity, such as air drag, Magnus force or wind currents. These forces deviate the trajectory from a perfect parabola compared to motion in a vacuum in astonishing ways (McBeath et al., 2008). For instance, previous works indicate that air drag can reduce flight time and distance traveled by a flying ball up to 50% (Brancazio, 1985; Adair, 2002). Therefore, trajectories initially on a collision course with the observer are no longer so after a short period. This pattern would potentially preclude the use of different algorithms for estimating the TTC, such as the Tau or GS model.

It has been argued that the self-regulatory nature of information-based strategies can efficiently deal with dynamic effects in a parabolic trajectory, provided that continuous visual information is available. In contrast, it is commonly argued that an internal model assuming a constant gravitational acceleration would be insufficient to account for dynamic forces such as air drag (Fink et al., 2009). To account for air drag, an internal model would have to gain access to a drag coefficient, mass and size for every single object and environment dynamically, which limits a massive application (Craig et al., 2006). Furthermore, it seems at odds with the fact that most people think that objects fall at the same rate despite their mass or volume (Oberle et al., 2005). However, explicit knowledge of physics may not affect performance in action-related tasks (Reed et al., 2010; Flavell, 2014). Following this reasoning, in our view, predictions using priors would only include variables facilitating the interpretation of the most generic case of natural law or parameters for a given task. In the following, we will show how the GS model, which relies only on gravity and size priors, can predict the remaining TTC reliably for the general case of trajectories under gravity and air drag conditions.

Unlike the gravitational force, which exerts the same force for different projectiles, air resistance depends on several factors: *ρ*, cosity of the environment surrounding the object; *C*_d_, a drag coefficient relative to the texture and shape of the projectile essentially; r object’s radius and v, the tangential speed of the object estimated dynamically. To simulate the effects of air drag on a parabolic trajectory, we followed the procedure described in Timmerman and van der Weele (1999) and Gómez and López-Moliner (2013). We simulated different trajectories under two different conditions: gravity only and gravity + air drag. Air viscosity around the ball (*ρ*) was set to 1.225 *kg/m*^3^ (value at sea level), and *C*_d_ was set to 0.346 or 0.4 for baseball and soccer balls, respectively (Alam et al., 2010; Kagan and Nathan, 2014). We introduced one initial vertical speed *V_y0_* = 9.807 m/s (2 s of flight time under gravity only conditions) for each trajectory and corresponding approaching speeds (*V_y0_* = 7.5,15,25 m/s) different balls launched at the origin. In this simulation, we did not include horizontal displacements.

**Figure 9:**
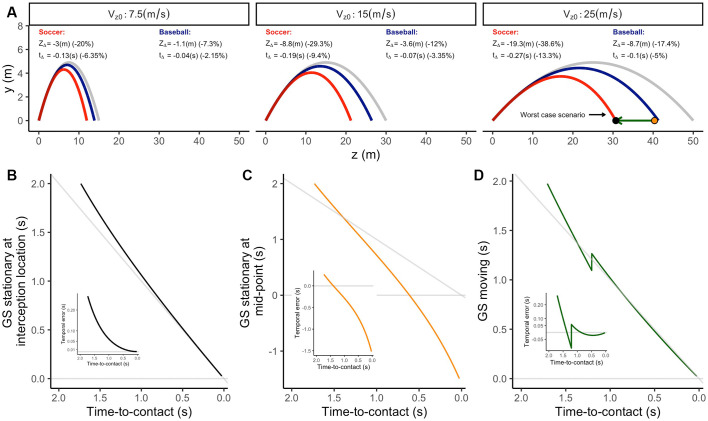
**(A)** Lateral view of different parabolic trajectories under gravity (gray lines) and gravity + air drag conditions for two different balls (red: soccer ball; blue: baseballs) and three initial approaching speeds (different panels). The figure annotates the difference in distance traveled (*Z*_Δ_) and flight duration (*t*_Δ_) compared to a trajectory only considering gravity. The black and orange dots in the third panel indicate the position of the corresponding simulated observer in panels **(B)** or **(C,D)**, respectively. The green arrow indicates the displacement simulated in panel **(D)**. Panels **(B–D)** indicate the predicted TTC using the GS model for different simulated observers in the worst-case scenario simulated. Insets depict the corresponding temporal errors using the predictions of the GS model.

In Figure 9A, the reader can see how air drag influences the trajectory described by the ball in both: the spatial and the temporal domain. In Figure 9A, the gray trajectory represents the trajectory followed by a ball under gravity-only conditions, whereas the blue and red trajectories indicate the trajectories followed by baseball and soccer balls including air drag in the simulation. For soccer balls, the effect of air drag is more pronounced mainly due to a larger cross-sectional area against the air. Note that since the initial vertical speed was the same for all the trajectories, the differences in flight time and distance traveled can be attributed to the different approaching speeds.

Thus, how well can the GS model estimate the remaining TTC in trajectories, including air drag? To answer this question, we simulated the output of the GS model for the worst-case scenario previously simulated. In that case, the GS model will yield the least accurate predictions. As shown in Figure 9A, the trajectory most affected by air drag is when a Soccer ball moves at the highest horizontal speed (*V_z0_* = 25 m/s).

To test the model’s accuracy, we used three different situations. In the first one, the observer is stationary at the interception position under gravity + air drag conditions (black dot in Figure 9A). In the second, the observer is stationary at a midpoint between the fall point under gravity-only and gravity + air drag conditions (black dot in Figure 9B). Finally, we simulated a situation in which the observer is at the same “mid-point.” However, in this case, the observer moves towards the intercept point at a constant speed (8.13 m/s), 500 ms after motion onset (green arrow in Figure 9A).

The output of the model for corresponding situations is depicted in Figures 9B–D. In all cases, the GS model reflects initial temporal errors corresponding to the difference in flight time between trajectories under gravity-only and gravity + air drag conditions (see annotations within Figure 9A). When the observer stands still in the interception location (Figure 9B), the GS model presents a high degree of accuracy during most of the trajectory. For instance, 0.5 s before the collision, the output converges to temporal errors of about 10–20 ms. However, if the observer stands still at the midpoint (Figure 9C), the model’s output deviates severely. In this case, the rate of change in the predicted TTC remains consistently lower than −1. In principle, this pattern could inform the observer that the interception point would be ahead of their position. In contrast, when the observer heads towards the interception location (Figure 9D), the model yield accurate predictions.

These simulations provide evidence that the output of the information included within the GS model provides accurate and actionable predictions of the remaining TTC when the observer remains stationary in the interception location or displaces towards the interception location. Therefore, it could be used as a navigational strategy or to plan the final interceptive action even when air drag is present.

### Benefits and Limitations of the Generalization of the GS Model

The GS model, like Tau, provides temporal information that may involve certain predictive benefits compared to the error-nulling strategies within the outfielder problem. Nevertheless, it also has some limitations that will be addressed in this section.

First, the GS model is much more robust to sensory noise than Tau (Gómez and López-Moliner, 2013). It uses the rate of change of the elevation angle ($\dot{\gamma }$) to estimate the TTC instead of a much noisier variable, the rate of expansion ($\dot{\delta }$) upon which Tau relies.

Second, Tau only provides accurate estimates when the ball is moving at constant speed towards the observer. In contrast, the GS provides accurate estimates at launch independently of the observer’s position, which would provide an initial accurate temporal information useful for planning the action.

Third, the GS model overestimates the TTC for trajectories under air drag as a function of the difference in flight time between trajectories under gravity-only and gravity + air drag conditions. However, our simulations show that in combination with the observer movement, temporal errors of less than 50 ms are possible 1 s before collision (Figure 9D).

Fourth, in contrast with previous error-nulling strategies, the GS model provides temporal information and can help compensate for the temporal delays and occlusions due to its predictive nature. The TTC can be used to adjust locomotion speed, inform of the remaining TTC under restricted visibility conditions or plan the final manual interception. On its part, the rate of change of TTC could be used to adjust the direction of movement. In this sense, both signals are complementary. However, the viability of the latter requires being able to detect changes in the rate of change of the predicted TTC, which needs further research. Furthermore, the detection of variations in the rate of change of TTC will likely incur delays. Therefore, future studies should study to which extent the simultaneous TTC signal can compensate for these delays.

Finally, the prediction from the GS model shares some limitations with Tau; that is, dealing with non-spherical objects such as Rugby balls or Frisbees would need further elaboration. However, some studies found that those errors can be canceled out by adding binocular information at the latter stages of catching (Gray and Regan, 1998).

## Evidence of Prediction in Eye Behavior and Manual Interception

The main problem to find support for model-based controlled behavior is that, when possible, the observer would keep track of the trajectory continuously (Oudejans et al., 1999; Postma et al., 2014). Indeed, this is the case of the outfielder problem, for which there is only anecdotal evidence of successful catchers directing away the gaze from the ball (Chodosh et al., 1995). In this context, accurate actions would not allow us to discriminate between information-based and model-based control directly (Belousov et al., 2016). Because of that, we need to scrutinize scenarios in which simple solutions such as heuristics or mappings between sensory information and temporal correlates for temporal estimation or action initiation are not available (Zhao and Warren, 2015).

One possibility to unveil the need for prediction in action control is to manipulate the target’s visibility. It is the most widely used experimental manipulation to study the predictive nature of behavior in interception (Sharp and Whiting, 1974; Whiting and Sharp, 1974; López-Moliner et al., 2010; Brenner and Smeets, 2011; Spering et al., 2011; de la Malla and López-Moliner, 2015). Nevertheless, more natural conditions are essential to understand how an observer could use temporal estimates to guide their action. In natural conditions, our gaze is often shifted to different locations to gather the information that may be relevant shortly (Hayhoe et al., 2005). In other contexts, a player would divert her gaze to check for deviations caused by balls’ bouncing (Diaz et al., 2013) or confirm whether the ball was appropriately hit (Mann et al., 2013). Some studies in manual interception and temporal estimations gave the observer complete freedom to decide which part of a trajectory they wanted to exploit visually while dealing with alternative tasks (Faisal and Wolpert, 2009; López-Moliner and Brenner, 2016; Aguado and López-Moliner, 2021). In those cases, where and when the observer averts the gaze from the target may provide valuable clues about the most relevant pieces of information according to task demands. Therefore, future studies might investigate when people prefer to divert the gaze from the ball while moving towards the interception location.

In some cases, it has been suggested the existence of privileged portions of the trajectory available for an observer to judge TTC. For example, in juggling or catching a ball, looking at the apex would provide the most relevant information (Whiting, 1968; Todd, 1981; Watson et al., 1992). However, a closer look at experimental data indicates that an observer does not actively search for a particular position in the parabola. Instead, prefers to use fixed temporal viewing windows generating priors during the task. These priors could then be used to weight visual information or estimate TTC when sensory information is unavailable (Amazeen et al., 1999; López-Moliner and Keil, 2012; Aguado and López-Moliner, 2021).

For example, most studies show acceptable catching performance in manual interception tasks for short flight durations no matter the section of the trajectory viewed. However, visual information had to be captured at least 200 ms before the catch to avoid sensorimotor delays (Sharp and Whiting, 1974; López-Moliner et al., 2010; López-Moliner and Brenner, 2016). For longer flight durations (up to 2 s), catching performance describes an inverted U shape with respect to flight duration (Sharp and Whiting, 1974; Amazeen et al., 1999). If the observer can only see the ball well in advance, performance would be low because the predictions decay rapidly (Binsted et al., 2006; Aguado and López-Moliner, 2021).

Zhao and Warren (2015) reasoned that in the case of short flight durations, part of an observer’s performance could be explained by the observer having learnt some of the regularities of a predictable trajectory mapping optic variables with a temporal correlate. Still, this would indicate the usefulness of developing priors during the task, which would be exploited when online visual information is not available. However, the fact that an observer exploits visual information when optic mappings are available indicates that they prefer to update their predictions based on the latest available visual information and combine it with evidence from previous knowledge (Mazyn et al., 2007; Binaee and Diaz, 2019). In the end, having a rough prediction is better than none (Brenner and Smeets, 2018).

In this line, de la Malla and López-Moliner (2015) proved that general rules of integration apply to the estimation of the TTC, which means: the observer integrates past and concurrent information to optimize the precision of temporal responses in a continuous fashion (Todorov, 2004; Liu and Todorov, 2007; Dimitriou et al., 2013). Assuming this is true, we can use Kalman filters to predict an observer’s estimation of TTC and response variability. A Kalman filter (Kalman, 1960) is a Bayesian tool that estimates the state of a system combining new noisy estimates, a prediction from prior measurements and a prior knowledge of how the system behaves. Using this technique, we could estimate both the accuracy and precision of online measurements for temporal judgments, manual interceptive tasks, and more general interceptive tasks such as the locomotion within the outfielder problem.

## Future Research

One of the main objectives of this work is to highlight the potential role of prior knowledge in calibrating visual information in terms of actionable predictions such as TTC. In our view, drawing predictions based on prior knowledge is not just a reliable and accurate way to predict future states of the environment but also helps us override the need to use unreliable optic cues (Cutting and Vishton, 1995). For instance: expansion rate ($\dot{\delta }$) and thus Tau (*τ*) might not be available at large distances, optic acceleration ($\overset{˙˙}{\gamma }$) is hardly discriminable by humans and would not allow for the continuous control of action (Werkhoven et al., 1992) even though some studies claim that the values of optical acceleration available are large enough to be detected (Babler and Dannemiller, 1993; Zaal et al., 2012); lastly, the literature is mixed with regards to the benefits of providing binocular disparity. Because of these reasons, here we advocate using the rate of change in the elevation angle ($\dot{\gamma }$), which is very precise, in combination with an internalized knowledge of gravity and physical size for the estimation of TTC in the GS model.

Here, the GS model provides different contexts to test the information included. For example, within the GS model, each contextual piece of information, gravity or size, is either in the denominator or the numerator. Hence, introducing proportional changes in the parameters governing the trajectory would result in proportional errors in the estimates of the remaining TTC. In a similar line, Jörges and López-Moliner (2017) showed that an observer might be able to extract information about the approaching speed of a ball through estimations of the rate of change of the elevation angle prior knowledge of gravity. Therefore, different values of gravity governing a trajectory should influence the prediction of the interception location.

Moreover, using TTC discrimination tasks, it could be possible to study if an observer can detect differences between trajectories under gravity-only conditions and gravity + air drag conditions. Our simulations indicated a WF of about 7% for the GS output (see Figure 7). Therefore, the difference in TTC should be above the discrimination threshold in some cases, as depicted in Figure 9A. Furthermore, decision tasks based on an observer’s ability to decide if there is enough time to perform alternate tasks (e.g., looking for teammates or running towards the interception area) from early visual information might be essential to test the availability of temporal estimates as a parameter to plan action for a broader range of interceptive actions.

On another note, it might be interesting to investigate the use of the GS model as a navigational mechanism. Since the GS model does not specify the interception location to plan interception in advance, we discovered a continuous coupling to keep a constant rate of change of the predicted TTC. To test if an observer would adapt locomotion to a constant rate of change, we should introduce players into contexts in which the value of gravitational acceleration or ball size do not correspond with the parameters assumed *a priori*. As introduced above, changes in the parameters would result in estimation errors of the remaining TTC. Thus, these manipulations would lead to predictions of the path followed by the observer. Nevertheless, to be able to use such a strategy, an observer might be able to detect deviations from different rates of change in TTC. To our knowledge, there is no previous work providing figures about how well people detect changes in TTC. Thus, our ability to detect differences in the rate of change and the time required to do so will need to be studied in future works.

To generate the suggested experiments, we need immersive and realistic spaces. Virtual scenarios will provide ecologically valid contexts to evaluate to what extent predictions influence interception. To do so, the use of wireless head-mounted displays (HMD) and portable eye-trackers will be essential. HMD insert the participants into rich and controlled environments already being used to train professional sports players (Zaal and Bootsma, 2011; Gray, 2017; Harris et al., 2020). Combining this technique with built-in eye-tracking systems provides access to how players interact with the environment to gather relevant visual information (Binaee et al., 2016; Moran et al., 2018). Those findings would still need to be replicated in real life under full-cue conditions. However, augmented reality devices are becoming more and more accessible and are likely to become more widespread. Those results may not be fully transferable to real life. However, it still would provide us information about human performance interacting with increasingly in-demand devices with potential applicability in a growing industry, eSports.

## Code Availability

The code to reproduce all the simulations included in this work can be found in the following link: https://osf.io/sa3cm/.

## Data Availability Statement

The datasets presented in this study can be found in online repositories. The names of the repository/repositories and accession number(s) can be found in the article.

## Author Contributions

JL-M initially conceptualized the manuscript. BA performed the literature review based on JL-M’s suggestions and initial guidance. BA and JL-M programmed the simulations. BA wrote the manuscript with input from JL-M. All authors contributed to the article and approved the submitted version.

## Conflict of Interest

The authors declare that the research was conducted in the absence of any commercial or financial relationships that could be construed as a potential conflict of interest.

## Publisher’s Note

All claims expressed in this article are solely those of the authors and do not necessarily represent those of their affiliated organizations, or those of the publisher, the editors and the reviewers. Any product that may be evaluated in this article, or claim that may be made by its manufacturer, is not guaranteed or endorsed by the publisher.
